# Intra-Rater and Inter-Rater Reliability of Tongue Coating Diagnosis in Traditional Chinese Medicine Using Smartphones: Quasi-Delphi Study

**DOI:** 10.2196/16018

**Published:** 2020-07-09

**Authors:** Zhi Chun Wang, Shi Ping Zhang, Pong Chi Yuen, Kam Wa Chan, Yi Yi Chan, Chun Hoi Cheung, Chi Ho Chow, Ka Kit Chua, Jun Hu, Zhichao Hu, Beini Lao, Chun Chuen Leung, Hong Li, Linda Zhong, Xusheng Liu, Yulong Liu, Zhenjie Liu, Xin Lun, Wei Mo, Sheung Yuen Siu, Zhoujian Xiong, Wing Fai Yeung, Run Yun Zhang, Xuebin Zhang

**Affiliations:** 1 School of Chinese Medicine Hong Kong Baptist University Hong Kong China (Hong Kong); 2 School of Computing Science Hong Kong Baptist University Hong Kong China (Hong Kong); 3 Department of Medicine The University of Hong Kong Hong Kong China (Hong Kong); 4 Shanghai Ninth People's Hospital Shanghai Jiao Tong University School of Medicine Shanghai China; 5 Guangdong Provincial Hospital of Traditional Chinese Medicine, The Second Affiliated Hospital of Guangzhou University of Traditional Chinese Medicine Guangzhou China; 6 Hong Zhi Tang Chinese Medicine Clinic Hong Kong China (Hong Kong); 7 Guang Dong Second Traditional Chinese Medicine Hospital Guangzhou China; 8 Yongan Hospital Foshan China; 9 School of Nursing Hong Kong Polytechnic University Hong Kong China (Hong Kong); 10 China Academy of Chinese Medical Sciences Guang An Men Hospital Beijing China

**Keywords:** mobile health, smartphone, traditional Chinese medicine, telemedicine, tongue image, machine learning, oral disease, Gwet AC2, COVID-19

## Abstract

**Background:**

There is a growing trend in the use of mobile health (mHealth) technologies in traditional Chinese medicine (TCM) and telemedicine, especially during the coronavirus disease (COVID-19) outbreak. Tongue diagnosis is an important component of TCM, but also plays a role in Western medicine, for example in dermatology. However, the procedure of obtaining tongue images has not been standardized and the reliability of tongue diagnosis by smartphone tongue images has yet to be evaluated.

**Objective:**

The first objective of this study was to develop an operating classification scheme for tongue coating diagnosis. The second and main objective of this study was to determine the intra-rater and inter-rater reliability of tongue coating diagnosis using the operating classification scheme.

**Methods:**

An operating classification scheme for tongue coating was developed using a stepwise approach and a quasi-Delphi method. First, tongue images (n=2023) were analyzed by 2 groups of assessors to develop the operating classification scheme for tongue coating diagnosis. Based on clinicians’ (n=17) own interpretations as well as their use of the operating classification scheme, the results of tongue diagnosis on a representative tongue image set (n=24) were compared. After gathering consensus for the operating classification scheme, the clinicians were instructed to use the scheme to assess tongue features of their patients under direct visual inspection. At the same time, the clinicians took tongue images of the patients with smartphones and assessed tongue features observed in the smartphone image using the same classification scheme. The intra-rater agreements of these two assessments were calculated to determine which features of tongue coating were better retained by the image. Using the finalized operating classification scheme, clinicians in the study group assessed representative tongue images (n=24) that they had taken, and the intra-rater and inter-rater reliability of their assessments was evaluated.

**Results:**

Intra-rater agreement between direct subject inspection and tongue image inspection was good to very good (Cohen κ range 0.69-1.0). Additionally, when comparing the assessment of tongue images on different days, intra-rater reliability was good to very good (κ range 0.7-1.0), except for the color of the tongue body (κ=0.22) and slippery tongue fur (κ=0.1). Inter-rater reliability was moderate for tongue coating (Gwet AC2 range 0.49-0.55), and fair for color and other features of the tongue body (Gwet AC2=0.34).

**Conclusions:**

Taken together, our study has shown that tongue images collected via smartphone contain some reliable features, including tongue coating, that can be used in mHealth analysis. Our findings thus support the use of smartphones in telemedicine for detecting changes in tongue coating.

## Introduction

Traditional Chinese medicine (TCM) has been practiced for over 3000 years in China and it has recently embraced mobile health (mHealth) [1-3]. In TCM, the practitioners combine tongue diagnosis with other signs and symptoms collected through the 4 diagnostic methods to attain a holistic view of the patient’s health status, so as to formulate strategies to adjust any imbalance in body functions [4]. Particularly, the color and texture of tongue coating or fur serves as a simple and effective means to aid syndrome differentiation in TCM [5]. Thus, tongue fur has been shown to be of significant importance in differentiation of TCM patterns associated with many diseases, such as asthma [6], gastric cancer [7], metabolic syndrome [8], hepatitis [9], gastritis [5], and, most recently, coronavirus disease (COVID-19) [10].

Anatomically, the dorsal surface of the tongue is divided by the sulcus terminalis into the anterior two-thirds (oral part), and the posterior one-third (pharyngeal part). TCM tongue diagnosis examines mainly the oral part, which is covered by a connective tissue core with overlying stratified squamous epithelium on the surface. The epithelium on the oral part of the tongue forms three types of papillae, and they have been named for their appearance: filiform, fungiform, and foliate papillae. In TCM, tongue fur refers to the keratinized tip of filiform papillae and dead epithelial cells, and its appearance is also affected by oral bacteria, blood metabolites, and salivary secretions from mucous and serous glands [11]. The fungiform papillae are highly vascularized and they provide the base color of the tongue body in TCM diagnosis. The foliate papillae are located at the edge of the tongue posteriorly and have an insignificant contribution to tongue coating.

In general, TCM diagnosis, including tongue diagnosis, has been found to be subjective with low inter-rater reliability [12-15]. Diagnosis made from tongue images collected by mobile devices may be more variable, due to variations in photographic techniques, camera settings, environmental lighting, light sources, display devices, etc [16]. These variations present barriers in the direct use of smartphone-collected tongue images in telemedicine and the application of machine learning for automatic tongue diagnosis in mHealth. Previously, we compared the results of tongue diagnosis from smartphone images and a commercially available tongue diagnostic device with those from direct inspection of human subjects. We found that the colors of tongue coating were the most consistently rated features between image inspection and direct subject inspection [17]. To further explore the characteristics of tongue diagnosis using smartphones, we set out to investigate the intra-rater and inter-rater reliability of tongue diagnosis using smartphone images. We first developed an operating classification scheme for tongue coating using a stepwise approach and a quasi-Delphi method. Subsequently, the study group used the same scheme to assess tongue features under direct subject inspection and with the tongue images of the same subjects. This was done to determine the reliability of tongue images in displaying features of the tongue. Finally, the intra-rater and inter-rater agreement of a representative tongue image set were evaluated to determine the reliability of tongue diagnosis using smartphone images. We hypothesized that certain tongue features would be recognized more consistently than others when examined through smartphone images.

## Methods

### Participants

**Figure 1:**
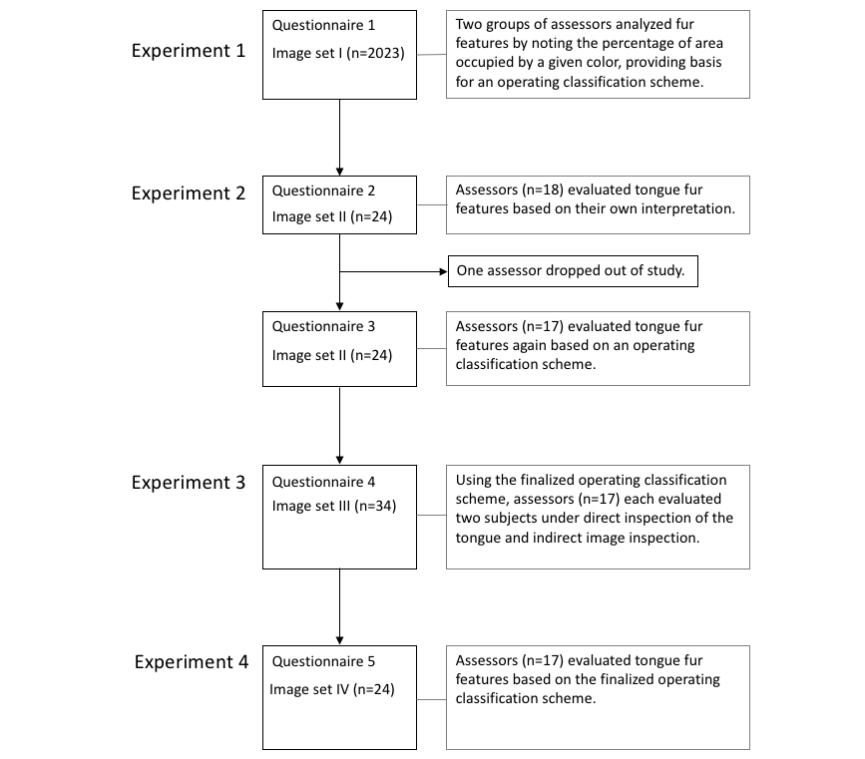
Flowchart of the study.

First, 2 groups of TCM students (n=2 in each group) who had completed a TCM diagnostic course were employed to work independently on the initial classification of Image Set I in Experiment 1. To develop a working classification scheme, a total of 18 TCM clinicians, all of whom were qualified Chinese medicine practitioners, participated in a quasi-Delphi process, which involved Experiments 2 and 3 (Figure 1). The Delphi process group of clinicians were from several university clinics in Hong Kong and their respective teaching hospitals in mainland China, or were research collaborators associated with these universities, with a median of 14 years in clinical practice (95% CI 12-23). The same group of clinicians participated in the assessment of intra-rater and inter-rater reliability.

### Image Collection

The study protocols for tongue image collection were designed in accordance with the Declaration of Helsinki and approved by the Committee on the Use of Human and Animal Subjects in Teaching and Research of Hong Kong Baptist University. Verbal informed consent was obtained from subjects after the nature of the study was explained. Tongue images were collected in various Chinese medicine clinics of Hong Kong Baptist University and its teaching hospitals in Guangzhou. Image set I consists of a total of 2023 tongue images taken with smartphones (iPhone [Apple Inc], HUAWEI [Huawei Technologies Co Ltd], Samsung [Samsung Group], and Xiaomi [Xiaomi Corp]; about 400 images per brand) and a computer-aided diagnostic system (DS01-A, Shanghai Daosheng Medical Technology Company; n=398) for previous studies [17]. Image set II consists of 24 representative images chosen from image set I. Image set III consists of 34 tongue pictures taken by 17 research participants using their own smartphones according to the standardized photographic protocol, and image set IV consists of 24 representative images chosen from image set III. The assessment of tongue image features was conducted online using the questionnaire builder Qualtrics to allow anonymous contributions by different participants from different locations (Figure 1).

### Standardized Photographic Protocol

Images in image set III and IV were collected using a standardized photographic protocol, which consisted of the following instructions: (1) start the phone’s camera function without using any filter or artificial intelligence (AI) function; (2), turn on the flash; (3) holding the phone directly in front of the subject, aim at their lips with a 45 degree angle with the phone 15-20 cm away; (4) tap on the screen to adjust focus when the subject protrudes the tongue and then take a picture; and (5) upload the full resolution image to the online questionnaire system. A 1-minute video demonstrating the photo-taking process was made available to all participants and written instructions were provided to indicate the resolution and orientation requirements for tongue images. The resultant images were checked for focus and orientation before being accepted into the study set. The images chosen for image set III were taken with iPhone (n=18), HUAWEI (n=11), and Xiaomi (n=5) smartphones.

### Study Flow

To develop the operating classification scheme for tongue fur, 2 groups of assessors in Experiment 1 independently analyzed image set I for fur features by noting the percentage of area that was covered by one of the designated colors of fur (ie, white, yellow, gray, and black), without specifying a designated fur feature. They also analyzed tongue fur textures based on the standard description by the World Health Organization (WHO) [18]. Based on the results from Experiment 1, an operating classification scheme was formed for later experiments. In Experiment 2, 18 raters determined tongue fur features in image set II based on their own interpretation first, then again based on the operating classification scheme. The inter-rater agreements of the two assessments were compared to see if the results of the working classification scheme were comparable with conventional judgements. Additional feedback from participants was also taken into consideration for improving the scheme. For efficiency reasons, we only examined tongue fur in Experiment 2 because other tongue features, such as shape and color of the tongue body, were known to be highly variable when judged by images [17,19]. In Experiment 3, the operating classification scheme was modified based on feedback and this classification scheme was used to assess the intra-rater agreement between direct subject inspection and indirect image inspection using image set III. Participants were instructed to first observe the subject directly and then record their observations of tongue features on the online questionnaire. They then took a picture of the subject’s tongue according to the standardized photographic protocol described above. Following that, they uploaded the image to the online questionnaire and answered the questions to describe the tongue features again based on their observation of the image. They were specifically instructed to not refer back to the rating based on direct inspection and to instead provide a new rating based on observation of the tongue image. In Experiment 4, which was conducted 24 hours after Experiment 3, the inter-rater agreement of image set IV was assessed with the operating classification scheme finalized by feedback from Experiment 3. The intra-rater agreement on tongue images between Experiments 3 and 4 was also assessed (Figure 1).

### Statistical Analysis

The data were entered into SPSS Statistics Version 25.0 (IBM), where analysis was conducted. Results were presented as Cohen k, chi-square and Gwet AC2. Cohen k was computed for intra-rater agreements and for agreement between 2 raters. When comparing agreements among multiple raters, percentage was used and chi-square was computed for statistical comparisons between groups. Furthermore, the value of Gwet AC2 was computed to indicate the extent of agreement among multiple raters [19,20].

## Results

### Experiment 1

**Figure 2:**
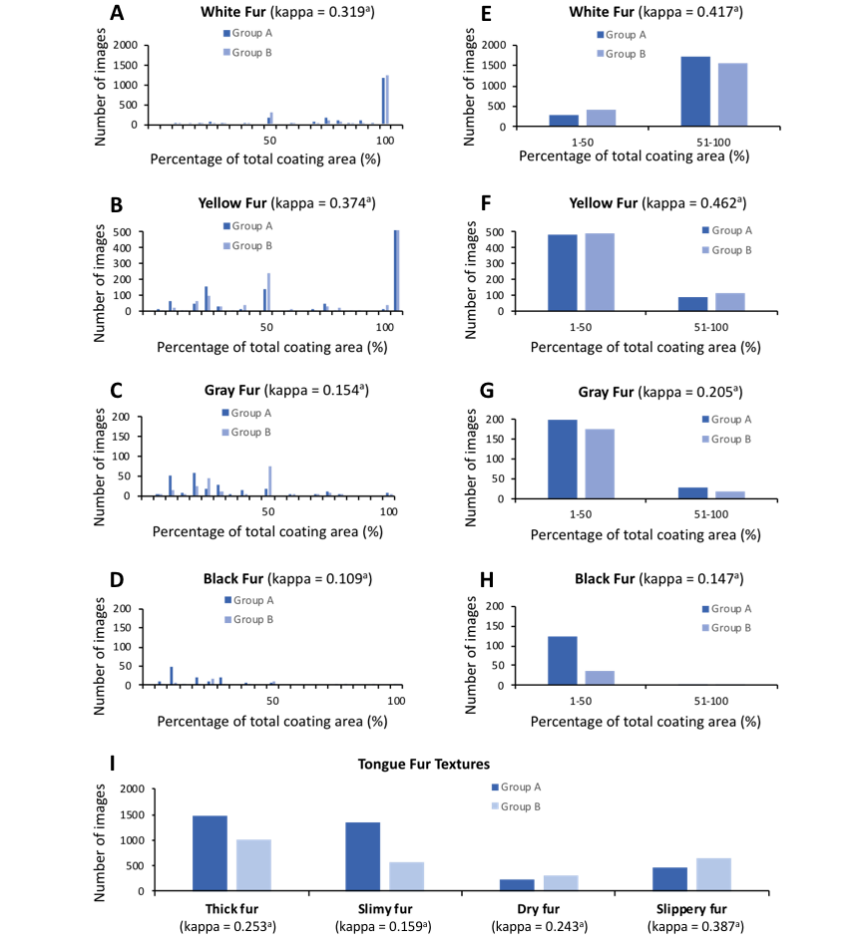
Results of tongue coating analysis on 2023 tongue images rated by 2 assessors. A-D) The original scoring results of white, yellow, gray, and black tongue fur, respectively. E-H) The scoring results after scores were sorted into two categories (1%-50% and 51%-100%). I) The results of tongue fur textures. The *P* value of κ is <.001.

Figure 2 shows the results of analysis of 2023 images by 2 groups of independent observers in Experiment 1. The percentage of area that is covered by one of the designated fur colors is shown in Figure 2A-D. We then combined the percentage of area values into two categories: those between 1% and 50% and those over 50%. As expected, the κ values for the 2 observers increased after combining the percentages (Figure 2E-H).

Based on findings from Experiment 1, an operating classification scheme using a broad description for the percentage of coverage that determined the color of tongue fur was developed; Table 1 displays the finalized version of the scheme.

**Table 1:** Tongue fur feature definitions and finalized operating classification scheme.

Tongue fur features	World Health Organization (WHO) definition	Finalized operating classification scheme
White fur	Tongue coating white in color	Over 50% of the total area covered by coating is white, with the remaining coating showing no prominent coloring.
Yellow fur	Tongue coating yellow in color	Over 50% of the total area covered by coating is yellow, with the remaining coating showing no prominent coloring.
Gray fur	Tongue coating gray in color	Over 50% of the total area covered by coating is light dark, with the remaining coating showing no prominent coloring.
Black fur	Tongue coating black in color	Over 50% of the total area covered by coating is black, with the remaining coating showing no prominent coloring.
Yellow and white fur	N/A^a^	There is prominent yellow coating occupying 50% or less of the total coating area, with the rest of the coating area being white or not obvious.
Gray and white fur	N/A	There is prominent light dark coating occupying 50% or less of the total coating area, with the rest of the coating area being white or not obvious.
Black and white fur	N/A	There is prominent black coating occupying 50% or less of the total coating area, with the rest of the coating area being white or not obvious.
Yellow and gray fur	N/A	There is yellow and light dark coating, with each color occupying 50% or less of the total coating area, with the rest of the coating area being white or not obvious.
Yellow and black fur	N/A	There is yellow and black coating, with each color occupying 50% or less of the total coating area, with the rest of the coating area being white or not obvious.
Gray and black fur	N/A	There is light dark and black coating, with each color occupying 50% or less of the total coating area, with the rest of the coating area being white or not obvious.
Yellow, gray and black fur	N/A	There is yellow, light dark, and black coating, with each color occupying 50% or less of the total coating area, with the rest of the coating area being white or not obvious.
Mirror tongue	A completely smooth tongue free of coating, like a mirror	Absence of any coating.
Thick fur	A tongue coating through which the underlying tongue surface is not visible.	Apart from the pharyngeal part of the tongue, there is an area covered by coating through which the underlying color of tongue body is not visible.
Slippery fur	A moist tongue coating with excessive fluid, feels slippery.	The tongue surface is moist with a more than normal amount of fluid.
Dry fur	A tongue coating that looks dry and feels dry to the touch	Tongue coating is dry with a less than normal amount of fluid.
Slimy fur	A dense, turbid, slimy tongue coating, sticking on the tongue, hard to wipe off.	There are areas where the coating particles are fine, dense, evenly formed, and tightly attached to the tongue.
Thin fur	A tongue coating through which the underlying tongue surface is faintly visible	Apart from the pharyngeal part of the tongue, the tongue is covered by coating through which the underlying color of tongue body is visible.

^a^N/A: not applicable.

### Experiment 2

Experiment 2 compared the results of tongue fur diagnosis based on the raters’ own interpretation versus those determined using the operating classification scheme on image set II (n=24). It can be seen from Table 2 that, with the exception of black fur, thin fur, and dry fur, similar percentages of inter-rater agreement were found for both diagnostic processes (*P*>.05, chi-square test), which indicates that the operating classification scheme is comparable with conventional judgements.

**Table 2:** Inter-rater agreements according to own interpretation (Questionnaire 2) or the operating classification scheme (Questionnaire 3) on image set II (n=24).

Category		Questionnaire 2			Questionnaire 3		
	Top 1 agreed features	Number of images	Average percentage of raters reaching agreement	Gwet AC2	Number of images	Average percentage of raters reaching agreement	Gwet AC2
**Color of fur**				**0.35**			**0.33**
	White	3	56		4	60	
	Yellow	4	57		9	61	
	Black	2	61		1	88^a^	
	Yellow and white	12	48		6	45	
	Yellow and black	2	50		4	44	
	Yellow and gray	1	44		1	30	
	Mirror tongue	2	86		3	91	
**Texture of fur**				**0.39**			**0.42**
	Thick	10	78		7	73	
	Slippery	5	77		4	78	
	Dry	4	68		N/A^b^	N/A	
	Slimy	17	76		12	69	
	Thin	4	82		7	66^c^	

^a^*P*=.04; chi-square test.

^b^N/A: not applicable.

^c^*P*=.01; chi-square test.

### Experiment 3

We slightly modified the operating classification scheme according to comments from participants in Experiment 2, and then applied it to the assessment of direct inspection and indirect image inspection for the same subject. Several participants reported that more details of tongue features could be recognized from smartphone images than from direct inspection, which supported the use of the standardized photographic protocol. As seen in Table 3, there was very good agreement between direct inspection and image inspection for color of tongue fur, thick fur, dry fur, slimy fur, thin fur, and teeth-marked tongue (κ range 0.85-1.0), and good agreement on slippery fur and color of the tongue body (κ range 0.69-0.70). These results indicate that smartphone photos taken with the standardized photographic protocol retain many features of tongue fur.

**Table 3:** Intra-rater agreements between direct tongue inspection and image inspection, and between the first and second image inspection.

Tongue features	Direct and image inspection, n=34		First and second image inspection, n=24		
	K value	*P* value		K value	*P* value
Color of tongue fur	0.89	<.001		0.70	<.001
Thick fur	0.94	<.001		0.83	<.001
Slippery fur	0.70	<.001		0.10	.62
Dry fur	0.85	<.001		0.80	<.001
Slimy fur	0.88	<.001		0.75	<.001
Thin fur	0.93	<.001		1.00	<.001
Teeth-marked tongue	1.00	<.001		0.83	<.001
Color of tongue body	0.69	<.001		0.22	.06

### Experiment 4

To allow the evaluation of inter-rater reliability using the finalized operating classification scheme, 24 representative images from image set III were selected to form image set IV (Table 4). The AC2 values for color of fur, texture of fur, color of tongue body, and tongue body feature were 0.49, 0.55, 0.34, 0.34, respectively. These findings indicate that there is a moderate level of inter-rater agreement on tongue fur features, but only a fair level of agreement on tongue body features.

**Table 4:** Inter-rater agreement based on the finalized operating classification scheme.

Category, Top 1 agreed features		Number of images	Average percentage of raters reaching agreement	Gwet AC2	
**Color of fur**					**0.49**
	White	7	65		
	Yellow	6	80		
	Yellow and white	4	50		
	Yellow and black	3	51		
	Yellow and gray	1	59		
	Yellow and gray and black	1	47		
	Mirror tongue	2	97		
**Texture of fur**					**0.55**
	Thick	16	83		
	Slippery	1	53		
	Dry	1	82		
	Slimy	16	86		
	Thin	6	78		
**Color of tongue body**					**0.34**
	Pale	4^a^	68		
	Pale red	15^a^	64		
	Red	7	52		
**Features of tongue body**					**0.34**
	Teeth-marked	8	83		
	Enlarged	7	71		

^a^In this instance, 2 images had the same number of raters choosing either Pale or Pale red as top 1 agreed feature in the category Color of tongue body, thus they were counted twice.

The intra-rater reliability on image set IV between the first and second assessments, separated by 24 hours or more, is shown in Table 3. It can be seen that the color of the tongue body and slippery fur showed no relationship between the assessments, whereas the other features show good to very good agreement.

## Discussion

### Principal Findings

To our knowledge, this is the first systematic study to evaluate intra- and inter-rater reliability of different tongue features using images from smartphones. Using a quasi-Delphi method, we developed an operating classification scheme to assess tongue fur features and then applied the scheme to direct tongue observation and indirect observation with tongue images. In addition, we have established a standardized photographic protocol for capturing tongue images with a smartphone. With the scheme and image collecting protocol in place, we found good to very good intra-rater agreement between the results of direct tongue inspection and those of smartphone images when rated immediately. This indicates that participants were generally satisfied with the quality of smartphone images in reflecting tongue features. However, when comparing the first and second assessments of the tongue images, the level of intra-subject agreement decreased, especially for the features of color of the tongue body and slippery fur. These results suggest that certain features are less reliable than others in tongue diagnosis using smartphone images. Finally, we found a moderate level of inter-rater agreement for tongue fur features and fair agreement for tongue body features.

Traditionally, tongue diagnosis is performed by direct visual inspection of the subject, but systematic evaluation of rater agreement by multiple practitioners on tongue features is difficult to achieve in clinical situations. It has been reported that agreement on overall TCM diagnoses is generally low; tongue diagnosis is one part of the criteria. Using tongue images, a number of studies examined different kinds of agreement in tongue diagnosis and the findings are quite variable, depending on the type of comparison [12,17,21]. Kim et al [21] found low levels of inter- and intra-rater practitioner agreements on tongue images and identified that inadequate operational definitions was the major contributing factor for the inconsistencies. To standardize operational definitions and procedures, in this study we developed an operating classification scheme and a standardized photographic protocol, which appear to have improved the level of inter-rater agreement, especially on tongue fur color. However, we found that the assessments of tongue body and slippery fur are unreliable, which is consistent with previous findings [15,17].

Previously, classification of tongue fur images has either been based on expert opinion or computer analysis, but no scheme has been accepted as the standard method. Zhen et al [19] recently studied 50 TCM experts and classified their diagnosis methods into 3 categories based on their internal consistency, external consistency, or both. This approach aimed to identify the experts who could provide the most consistent diagnosis. However, as the classification methods are not overtly stated, this approach remains a black-box approach. We are in the process of training AI for automatic tongue diagnosis, which requires labelling a large number of images for machine learning. For this purpose, it is not practical to find experts with consistent diagnosis skills to do the labelling task. Here we have employed a different approach, whereby we developed a simple classification scheme and evaluated the scheme as used by clinicians with different levels of experience. In so doing, we generated an operational framework and improved the definitions through feedback from participants. The advantage of our approach is that, once defined, the operating classification scheme can be used by any person with basic training in TCM tongue diagnosis to label tongue images, and the classification results are expected to produce similar inter-rater reliability.

### Strengths and Limitations

There are a number of strengths as well as limitations in this study. The first strength is in its novelty with the use of smartphones without any color correction measures. It is generally believed that the use of smartphones instead of a purpose-built tongue imaging system would produce unreliable results, due to variations in factors such as camera settings, light source, and display settings [16]. However, we demonstrated, for the first time, that using our tongue photographic protocol, it is possible to obtain good quality tongue images with a good to very good level of intra-rater agreement against direct tongue inspection, and with a moderate level of inter-rater agreement on fur features. In this regard, it is interesting to note that the level of inter-rater agreement achieved using smartphone images in our study is comparable with those findings obtained through direct inspection of the tongue [15]. On the other hand, we found a reduced intra-rater reliability between the first and second assessments on tongue images, which suggests that human factors also play an important role in the variability of tongue diagnosis.

Another strength of this study is its practically oriented design. We tested the photographic protocol on a number of smartphones under uncontrolled situations, using practitioners with differing levels of clinical experience and tongue images of various fur colors. In addition, we kept both the classification scheme and the photographic protocol simple and practical. We believe that tongue images collected and evaluated in this way would have good external validity, suitable for inspection by TCM practitioners or development of a diagnostic app using AI. It is also interesting to note that a number of Delphi participants commented that, compared with routine clinical tongue inspection, more details of tongue features can be recognized within smartphone images. In fact, smartphone-captured tongue images have been used extensively in the recent TCM management of COVID-19, and it was believed that tongue coating diagnoses played an important role in the formulation of TCM treatment strategies [10]. Taken together, smartphone tongue images may be used as an initial screening in areas of telemedicine other than TCM, including common tongue conditions [22,23], diabetes [24], cancer [25], and COVID-19 [26].

One of the limitations of this study is that we were not able to assess the level of inter-rater agreement on direct tongue inspection. This would have been useful as a comparison to that of tongue image inspection. However, it was not feasible to have many assessors examine 24 subjects face-to-face repeatedly. Another limitation of this study is that the total number of tongue images in the final set (image set IV) for assessment of inter-rater agreement was relatively small. We restricted image set IV to 24 images because we received feedback from assessors that too many images could lead to mental fatigue and inaccurate labelling. For future studies, it would be advisable to separate the image set into smaller sets if a larger number of images were used.

### Conclusions

By using a simple photographic protocol with smartphones, it is possible to obtain tongue images that contain many features of the tongue, especially those related to tongue coating. This study provides information on the inter-rater reliability of different tongue features, which can be used as future references for mHealth, including telemedicine by practitioners or automatic tongue diagnosis by AI. Taken together, our findings support the use of smartphone images for tongue coating analysis.

## Abbreviations

AI: artificial intelligence
COVID-19: coronavirus disease
mHealth: mobile health
TCM: traditional Chinese medicine
WHO: World Health Organization
